# Editor’s introduction to the special section on the 7th Biomedical Linked Annotation Hackathon (BLAH7)

**DOI:** 10.5808/gi.19.3.e1

**Published:** 2021-09-27

**Authors:** Jin-Dong Kim, Kevin Bretonnel Cohen, Fabio Rinaldi, Zhiyong Lu, Hyun-Seok Park

**Affiliations:** 1 Database Center for Life Science (DBCLS), Research Organization of Information and Systems (ROIS), Kashiwa, Chiba 277-0871, Japan; 2 School of Medicine, University of Colorado, Aurora, CO 80045, USA; 3 Dalle Molle Institute for Artificial Intelligence Research (IDSIA), 6928 Manno, Switzerland; 4 National Center for Biotechnology Information (NCBI), National Institutes of Health (NIH), Bethesda, MD 20894, USA; 5 Center for Convergence Research of Advanced Technologies, Ewha Womans University, Seoul 03760, Korea

The special section is dedicated to reporting achievements of the *7th Biomedical Linked Annotation Hackathon (BLAH7)*. *BLAH* is an annual hackathon event which is organized to join forces of biomedical text mining for the goal to promote interoperability among text mining resources. This year, the 7th edition was held in January, 2021. Due to the pandemic, it was organized as an online event, with the special theme “*coronavirus disease 2019 (COVID-19)*”. The goal was to develop text mining resources to help address the pandemic situation. During the hackathon, 47 participants from 11 countries worked on voluntarily organized projects, and the results are reported in this special collection.

This section includes seven application notes and one opinion article. The first application note by Hernandez et al. [1] presents a Twitter dataset which includes more than 120 million “potentially clinically-relevant” tweets. The tweets are automatically annotated for clinically important named entities like drugs and symptoms. The dataset is released publicly to facilitate research on mining social media data for biomedical and clinical applications. Lithgow-Serrano et al. [2] presents named entity annotation of the LitCovid [3] dataset using OntoGene’s Biomedical Entity Recogniser (OGER) [4] and shows its effectiveness for document classification. Ouyang et al. [5] presents the AGAC annotation [6] added on top of the PubTator [7] and OGER annotations and shows that the addition is potentially useful to mine regulatory or causal relationships between biomedical entities. The following three papers represent efforts for multilingualism of text mining. Barros et al. [8] presents a multilingual parallel corpus of PubMed articles for the language pairs English-Portuguese and English-Spanish. Their corpus was annotated for biomedical entities and also relationships between them, which was then used to develop a multilingual recommendation dataset for recommending biomedical entities to the authors of the articles. Yamaguchi et al. [9] and Soares et al. [10] are written by the same set of authors. They developed two versions of Japanese translation of MeSH terms, one through merging of existing resources and manual curation, and another through an automatic translation method, of which the results are reported in the two separate application notes. Larmande et al. [11] reports a revision to OryzaGP [12], a corpus of PubMed articles relevant to rice species, which are automatically annotated for proteins and genes. The last one by Dohi et al. [13] presents the authors’ opinion after their case study with Alexander disease towards visualizing the phenotype diversity.

Based on the spirit of sharing, most of the resulting datasets, including corpora, annotations, and dictionaries, are released through open repositories like GitHub, PubAnnotation/PubDictionaries [14], and so on. We hope that this special collection will be an opportunity for the readers of the journal *Genomics & Informatics* to get informed about recent biomedical text mining activities aimed at providing support in the current COVID-19 pandemic situation.

## References

[1] Hernandez LA, Callahan TJ, Banda JM (2021). A biomedically oriented automatically annotated Twitter COVID-19 dataset. Genomics Inform.

[2] Lithgow-Serrano O, Cornelius J, Kanjirangat V, Méndez-Cruz CF, Rinaldi F (2021). Improving classification of low-resource COVID-19 literature by using Named Entity Recognition. Genomics Inform.

[3] Chen Q, Allot A, Lu Z (2021). LitCovid: an open database of COVID-19 literature. Nucleic Acids Res.

[4] Basaldella M, Furrer L, Tasso C, Rinaldi F (2017). Entity recognition in the biomedical domain using a hybrid approach. J Biomed Semantics.

[5] Ouyang S, Wang Y, Zhou K, Xia J (2021). LitCovid-AGAC: cellular and molecular level annotation data set based on COVID-19. Genomics Inform.

[6] Wang Y, Zhou K, Kim JD, Cohen KB, Gachloo M, Ren Y, et al. An active gene annotation corpus and its application on anti-epilepsy drug discovery. In: IEEE International Conference on Bioinformatics and Biomedicine (BIBM), 2019 Nov 18-21, San Diego, CA, USA. New York: Institute of Electrical and Electronics Engineers, 2019

[7] Wei CH, Kao HY, Lu Z (2013). PubTator: a web-based text mining tool for assisting biocuration. Nucleic Acids Res.

[8] Barros M, Ruas P, Sousa D, Bangash AH, Couto FM (2021). COVID-19 recommender system based on an annotated multilingual corpus. Genomics Inform.

[9] Yamaguchi A, Takatsuki T, Tateisi Y, Soares F (2021). Constructing Japanese MeSH term dictionaries related to the COVID-19 literature. Genomics Inform.

[10] Soares F, Tateisi Y, Takatsuki T, Yamaguchi A (2021). O-JMeSH: creating a bilingual English-Japanese controlled vocabulary of MeSH UIDs through machine translation and mutual information. Genomics Inform.

[11] Larmande P, Liu Y, Yao X, Xia J (2021). OryzaGP 2021 update: a rice gene and protein dataset for named-entity recognition. Genomics Inform.

[12] Larmande P, Do H, Wang Y (2019). OryzaGP: rice gene and protein dataset for named-entity recognition. Genomics Inform.

[13] Dohi E, Bangash AH (2021). Visualizing the phenotype diversity: a case study of Alexander disease. Genomics Inform.

[14] Kim JD, Wang Y, Fujiwara T, Okuda S, Callahan TJ, Cohen KB (2019). Open Agile text mining for bioinformatics: the PubAnnotation ecosystem. Bioinformatics.

